# Digitalis Therapy Is Associated With an Increased Risk of ICD Shock Delivery and Device Revision

**DOI:** 10.1111/anec.70080

**Published:** 2025-04-06

**Authors:** Gerrit Frommeyer, Philipp S. Lange, Thomas Kleemann, Christoph Stellbrink, Hüseyin Ince, Johannes Brachmann, Thorsten Lewalter, Matthias Hochadel, Jochen Senges, Lars Eckardt

**Affiliations:** ^1^ Clinic for Cardiology II – Electrophysiology University of Münster Münster Germany; ^2^ Department of Cardiology Klinikum der Stadt Ludwigshafen Ludwigshafen Germany; ^3^ Department of Cardiology Klinikum Bielefeld Bielefeld Germany; ^4^ Department of Cardiology Vivantes Klinikum Am Urban and Neukölln Berlin Germany; ^5^ Rostock University Rostock Germany; ^6^ Medical School REGIOMED, University of Split School of Medicine Split Croatia; ^7^ Department of Medicine Osypka Herzzentrum Munich Germany; ^8^ Stiftung Institut für Herzinfarktforschung (IHF) Ludwigshafen Germany

**Keywords:** cardiac resynchronization therapy, digitalis, heart failure, implantable cardioverter/defibrillator

## Abstract

**Background:**

Digitalis glycosides are employed for rate control of atrial fibrillation and treatment of heart failure. Previous studies suggested potential harmful effects of digitalis therapy. The aim of the present study was to assess the prevalence and potential impact of digitalis therapy on outcomes in patients with systolic failure who were implanted with an ICD‐ or CRT‐ICD system.

**Methods and Results:**

The German Device Registry is a nationwide, prospective registry with a 1‐year follow‐up investigating 4384 patients receiving either ICD or CRT systems in 52 German centers. The present analysis focused on the presence of digitalis therapy in 3826 patients undergoing device implantation. Patients receiving digitalis therapy (*n* = 800) presented a more severely impaired left ventricular function, higher NYHA class, and an increased incidence of left bundle branch block. Consequently, the implantation of CRT systems was more common in this group. One‐year mortality did not significantly differ between both groups (9.1% vs. 7.4%, *p* = 0.14). Similar results were obtained for the combined endpoint, including death, myocardial infarction, and stroke. ICD shock delivery (19.7% vs. 15.0%, *p* = 0.006) and device revision (11.4% vs. 7.5%, *p* < 0.004) were more common in digitalis‐treated patients.

**Conclusion:**

In this study in patients undergoing ICD or CRT implantation, an association of digitalis therapy with an increased risk of device revision was observed. Of note, mortality or severe cardiovascular events did not differ between both groups. Furthermore, an increased risk of ICD shock delivery was observed in digitalis‐treated patients.

## Graphical abstract

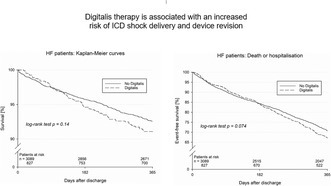

## 1 Introduction

The WHO continues listing digoxin as one of the essential antiarrhythmic and heart failure medicines. However, the true value of digitalis glycosides is under debate. For example, digitalis does not play a major role in contemporary European Heart Failure (McDonagh et al. 2021) or Atrial Fibrillation (AF) Guidelines (Hindricks et al. 2021). The current guidelines of the European Society of Cardiology (ESC) for the treatment of chronic heart failure indicate that digoxin may be considered in patients with heart failure with reduced left ventricular function, sinus rhythm, and impaired left ventricular function to reduce heart failure hospitalizations (McDonagh et al. 2021). These recommendations are based on older studies such as the Digitalis Investigation Group (DIG) trial, which has proven that digitalis did not affect overall mortality but was associated with an important reduction of hospitalizations (Ahmed et al. 2006; Digitalis Investigation Group 1997). Besides, the ESC guidelines for the management of atrial fibrillation (AF) suggest digoxin as a suitable option for rate control in patients with a left ventricular ejection fraction below 40% with the same level of evidence as for beta blockers (Hindricks et al. 2021). This accounts in particular for hemodynamically unstable patients, as digitalis glycosides are not associated with a relevant depression of blood pressure.

The recent Rate‐AF trial suggested at least no negative effects of low‐dose digoxin compared with bisoprolol in patients with permanent AF and symptoms of chronic heart failure in the presence of fewer adverse effects in the digoxin arm (Kotecha et al. 2020).

Of note, previous experimental and clinical data have suggested potential deleterious effects of digitalis therapy. The results of the PALLAS trial (Connolly et al. 2011), which examined the potential antiarrhythmic properties of dronedarone in heart failure patients with long‐standing persistent AF, displayed an increased mortality in dronedarone‐treated patients. This effect was at least partially explained by potential interactions between digitalis and dronedarone (Hohnloser et al. 2014).

Experimental data demonstrated an increased vulnerability mediated by significantly shortened refractory periods in an experimental model where combined treatment with dronedarone and digitalis glycosides was simulated (Frommeyer et al. 2015). Of note, amiodarone did not exert these effects in the same model (Frommeyer et al. 2017). Sole digitalis therapy was also associated with ventricular arrhythmias in the same model.

In this study, data from a multicenter real‐world registry on patients undergoing device implantation was analyzed to assess the potential consequences of digitalis therapy.

## 2 Methods

The German Device Registry is a nationwide, prospective database on patients who underwent implantation of implantable cardioverter‐defibrillators (ICD) or cardiac resynchronization therapy (CRT) systems in Germany. Data collection is organized by the Stiftung Institut für Herzinfarktforschung Ludwigshafen, Germany (IHF). Fifty voluntarily participating German centers committed themselves to include all consecutive consented patients between 2008 and 2015. The registry was approved by the local ethics committees. Details of the study design and procedures and overall results have been published previously (Bogossian et al. 2020; Frommeyer, Andresen, et al. 2019; Frommeyer et al. 2020).

This study includes patients undergoing implantation of ICD or CRT systems with a significant impairment of left ventricular function (defined as a left ventricular ejection fraction [LVEF]) below 40% and heart failure. The device implantation represented the index hospitalization. Follow‐up was scheduled prospectively at 1 year after device implantation by telephone and was conducted centrally by the IHF, resulting in a follow‐up duration of 12 months. Data on study endpoints was collected and analyzed after the implantation procedure, after discharge as well as after the described follow‐up period. During telephone contact, standardized questions on cardiac events (e.g., hospitalizations), complications, medication, heart failure symptoms, and patient satisfaction were discussed. In case of an ineffective call, further information was gathered from other caring physicians or civil registration offices.

Relevant study endpoints during hospital stay included death, non‐fatal myocardial infarction, non‐fatal stroke, and device revision. Endpoints during follow‐up were death, MACCE as a composite endpoint including death, myocardial infarction or stroke, re‐hospitalization, ICD shock delivery, and device revision.

Quality of life and patient satisfaction were assessed by posing a few specific questions during follow‐up. This included the question of whether therapy was regarded as successful, whether patients would decide again to undergo the procedure whether they felt effectively protected from potential sudden cardiac death, and whether they were afraid of a potential shock delivery.

### 2.1 Statistical Analysis

Continuous variables are presented as mean ± standard deviation. Categorical variables are expressed as the number and percentage of patients. Differences in categorical distributions were tested for statistical significance using chi‐squared tests. For binary variables, odds ratios with 95% confidence intervals were calculated. Rates of rare complications were compared using Fisher's exact test. The cumulative incidence of death and combined endpoints of death, myocardial infarction, and stroke during follow‐up at 366 days after index discharge was assessed using methods of survival analysis (Kaplan–Meier estimator, log‐rank test). In addition, we computed odds ratios by using multivariable logistic regression models adjusted for relevant risk factors (Table 1).

**TABLE 1:** Determinants for 1‐year mortality and ICD shock delivery (Cox‐regression).

Variable	*p*	Adjusted hazard ratio	95% CI
Digitalis	0.075	1.19	0.98–1.43
Age (per 10 years)	0.33	1.04	0.96–1.12
CRT‐D vs. ICD	< 0.001	0.73	0.60–0.88
NYHA III/IV	0.29	1.10	0.92–1.30
LVEF ≤ 30%	0.69	1.04	0.86–1.25
Coronary artery disease	0.25	1.11	0.93–1.32
AF at baseline	< 0.001	1.53	1.27–1.84

Abbreviations: AF = atrial fibrillation, LVEF = left ventricular ejection fraction.

*p* Values ≤ 0.05 were considered statistically significant. The statistics shown should be regarded as descriptive and were based on the available cases. All calculations were performed using the SAS 9.4 software package (SAS Institute, Cary, NC).

## 3 Results

### 3.1 Patient Characteristics/Demographics

Demographic characteristics are summarized in Table 2. Out of 4384 patients registered in the German Device Registry, 3826 patients met the inclusion criteria. Comparisons were made between patients with digitalis therapy at discharge (*n* = 800) and patients without digitalis therapy (*n* = 3026). There were no significant differences in age between digitalis‐treated patients and patients without digitalis therapy (*p* = 0.98). Dilated cardiomyopathy was more often present in digitalis‐treated patients (48.3%) as compared with controls (38.%, *p* < 0.001). Not surprisingly, this observation was reversed for coronary artery disease (67.1% in patients without digitalis vs. 55.4% in digitalis‐treated patients [*p* < 0.001]).

**TABLE 2:** Baseline patient characteristics and implantation procedure.

	Digitalis therapy	Control group	*p*
	*n* = 800	*n* = 3026	
Age [years]	66.8 ± 10.9	66.7 ± 11.4	0.98
Male [%]	84.0 (*n* = 672)	82.2 (*n* = 2487)	0.23
BMI	29.1	27.3	0.04
NYHA III+	63.1 (*n* = 483)	54.5 (*n* = 1594)	< 0.001
LVEF < 30% [%]	79.8 (*n* = 637)	73.0 (*n* = 2190)	< 0.001
LVEF < 25% [%]	51.6 (*n* = 375)	43.3 (*n* = 1186)	< 0.001
Coronary artery disease [%]	55.4 (*n* = 443)	67.1 (*n* = 2031)	< 0.001
Dilated Cardiomyopathy [%]	48.3 (*n* = 386)	38.7 (*n* = 1171)	< 0.001
LBBB [%]	44.4 (*n* = 355)	38.5 (*n* = 1164)	0.002
Atrial fibrillation [%]	35.9 (*n* = 287)	16.3 (*n* = 493)	< 0.001
Diabetes [%]	36.6 8 (*n* = 293)	30.2 (*n* = 913)	< 0.001
COPD [%]	4.6 (*n* = 37)	4.1 (*n* = 123)	0.48
CKD [%]	21.1 (*n* = 169)	20.2 (*n* = 610)	0.55
ICD (VVI) [%]	40.4 (*n* = 323)	46.1 (*n* = 1395)	0.004
ICD (DDD) [%]	12.8 (*n* = 102)	17.9 (*n* = 541)	< 0.001
CRT‐D [%]	46.8 (*n* = 375)	36.0 (*n* = 1090)	< 0.001
Primary prevention [%]	72.0 (*n* = 576)	70.5 (*n* = 2134)	0.41

Abbreviations: CKD = chronic kidney disease, COPD = chronic obstructive pulmonary disease, CRT = cardiac resynchronization therapy, ICD = implantable cardioverter/defibrillator, LBBB = left bundle branch block, LVEF = left ventricular ejection fraction.

Severe impairment of left ventricular function was more prevalent in digitalis‐treated patients (LVEF ≤ 30%: 79.8% vs. 73.0%, *p* < 0.001; LVEF ≤ 25%: 51.6% vs. 43.3%, *p* < 0.001). NYHA class III or worse was also more common in digitalis‐treated patients (63.1% vs. 54.5%, *p* < 0.001). Regarding comorbidities, there were no significant differences for hypertension, COPD, or renal impairment, while diabetes was observed more frequently in the digitalis group (36.6% vs. 30.2%, *p* < 0.001).

The analysis of the implanted device displayed more CRT‐D systems in digitalis‐treated patients (46.9% vs. 36.0%, *p* < 0.001). Consequently, VVI systems (40.4% vs. 46.1%, *p* = 0.003) and DDD systems (12.8% vs. 17.9%, *p* < 0.001) were implanted more often in patients without digitalis therapy. No significant differences were observed for primary or secondary prevention. Left bundle branch block was more common in digitalis‐treated patients (44.4% vs. 38.5%, *p* = 0.002). Atrial fibrillation was also present more often at the time of ECG recording in digitalis‐treated patients (35.9% vs. 16.3%, *p* < 0.001).

### 3.2 Implantation Procedure and Discharge

Device implantation was performed in 800 digitalis‐treated patients and in 3026 patients without digitalis therapy. Duration of implantation did not differ between both groups. A defibrillation test was performed in 72.8% of digitalis‐treated patients and in 74.5% of patients without digitalis therapy (*p* = 0.37). The success rate of defibrillation test did not differ between both groups (*p* = 0.13).

Regarding procedural in‐hospital complications, no significant differences were observed between both groups regarding death, nonfatal myocardial infarction, or nonfatal stroke. This also accounts for other complications, including pericardial effusion, pneumothorax, or pocket hematoma. Device revision before discharge was observed less frequently in digitalis‐treated patients (0.9% vs. 2.5%, *p* = 0.013).

Regarding medication at discharge, diuretics were prescribed more often in digitalis‐treated patients. In contrast, class‐III antiarrhythmic drugs were administered more often in patients not treated with digitalis. As a result of an increased incidence of AF, anticoagulation was more common in digitalis‐treated patients, whereas administration of platelet inhibitors was observed more frequently in patients without digitalis therapy. Differences regarding other cardiovascular medication are displayed in Table 3.

**TABLE 3:** Cardiovascular medication at time of index discharge.

	Digitalis group	Control group	*p*
ACE‐inhibitor/AT1 receptor antagonist [%]	92.3 (*n* = 738)	92.1 (*n* = 2786)	0.87
Betablocker [%]	92.0 (*n* = 736)	93.0 (*n* = 2814)	0.33
Aldosteron antagonist [%]	50.8 (*n* = 406)	449.7 (*n* = 1412)	0.039
Diuretics [%]	89.8 (*n* = 718)	79.3 (*n* = 2397)	< 0.001
Class‐III antiarrhythmic drugs [%]	10.5 (*n* = 84)	13.7 (*n* = 413)	0.018
Statin [%]	58.0 (*n* = 464)	64.7 (*n* = 1957)	< 0.001

### 3.3 Follow‐Up

Follow‐up information was obtained for 97.1% of patients in the digitalis group and 96.9% of patients without digitalis treatment. Mean follow‐up duration was 16.2 (12.9; 23.6) and 16.7 (13.1; 22.4) months, respectively.

**FIGURE 1:**
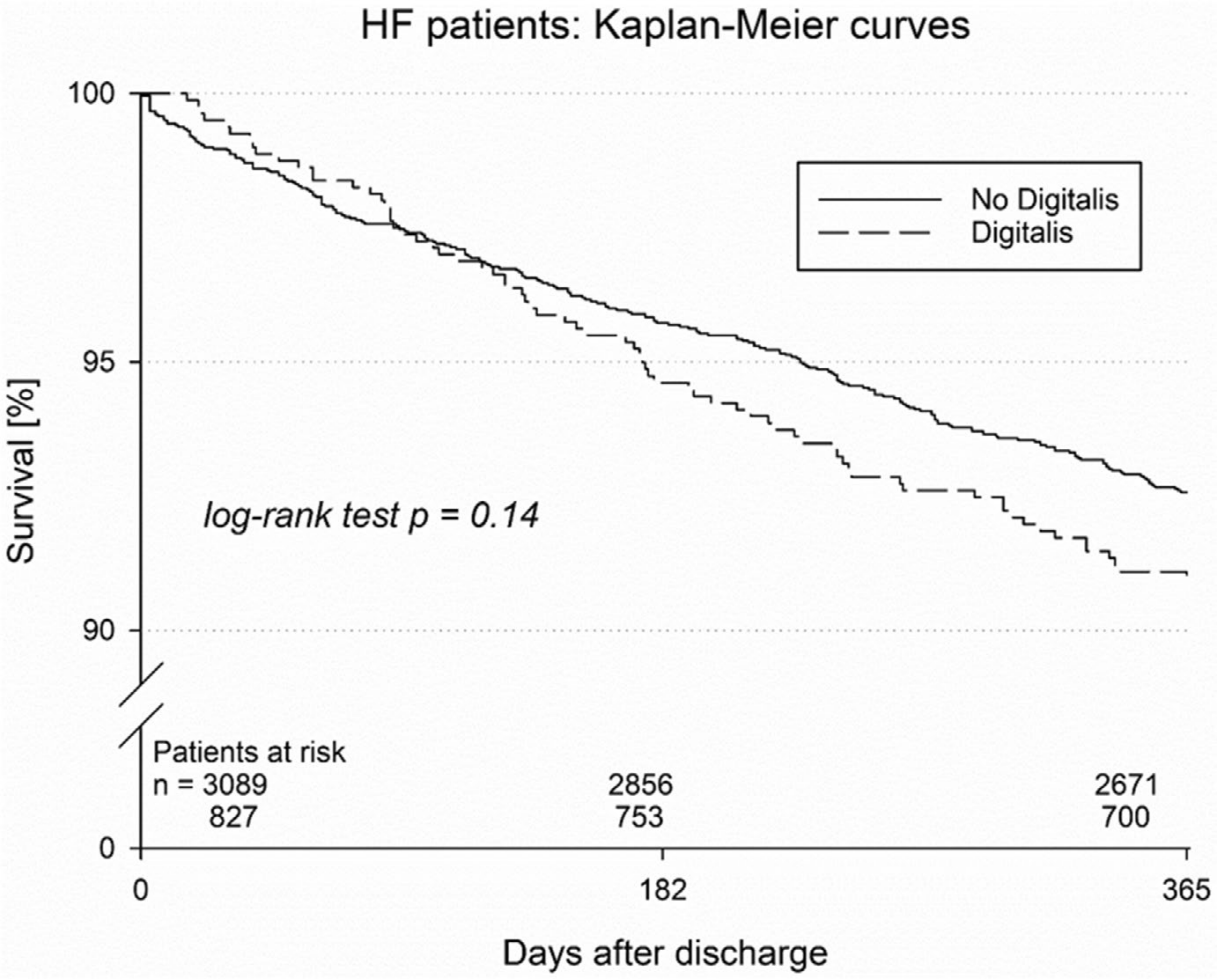
Kaplan–Meier curve for mortality in patients with and without digitalis therapy.

**FIGURE 2:**
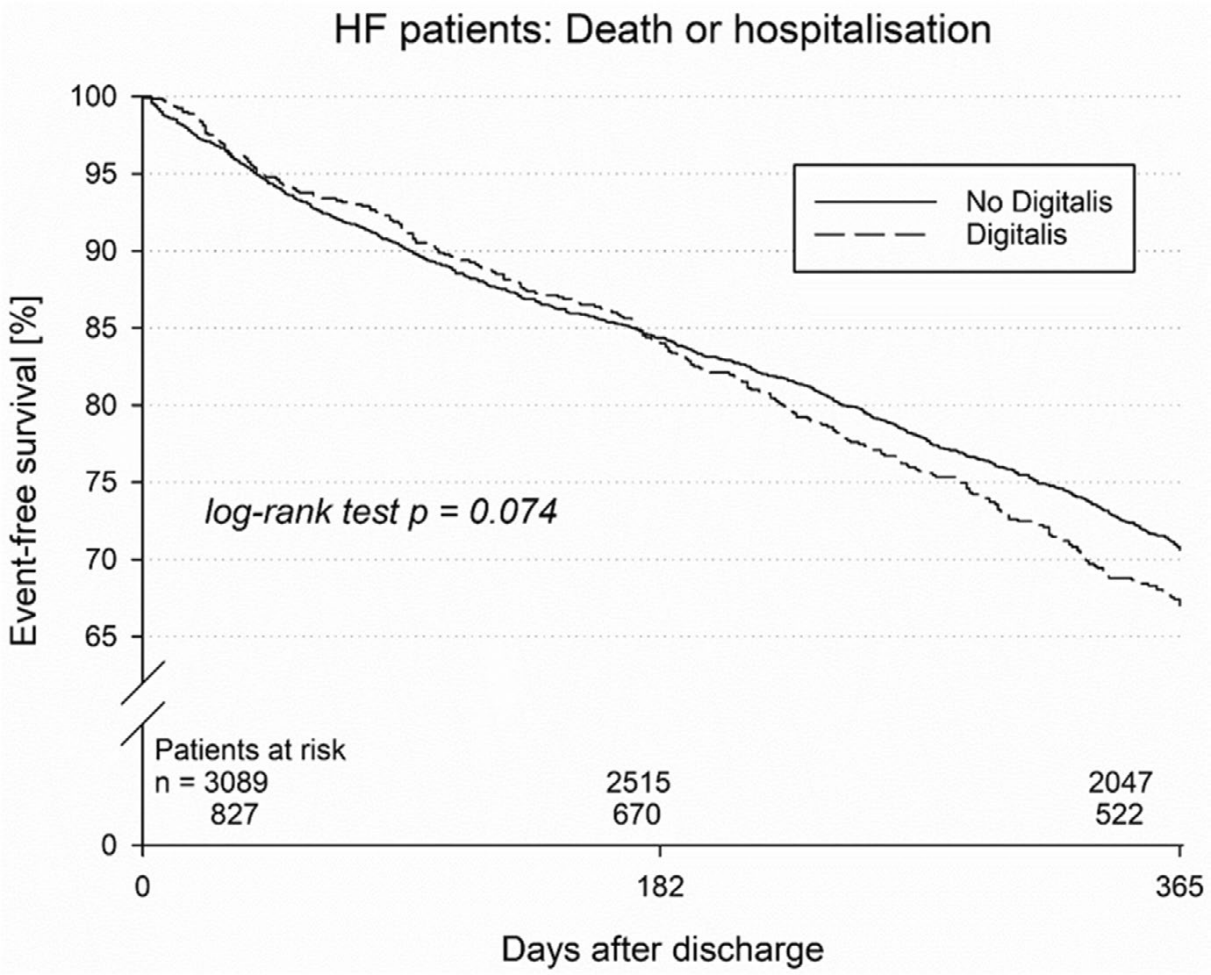
Kaplan–Meier curve for the combined endpoint of mortality and re‐hospitalization in patients with and without digitalis therapy.

The Kaplan–Meier estimate of 1‐year mortality was not significantly different and was estimated at 9.1% for digitalis‐treated patients versus 7.4% for patients without digitalis therapy (*p* = 0.14, Figure 1). Similar results were observed for the combined endpoint of death, myocardial infarction, or stroke (10.4% vs. 8.4%, *p* = 0.080, Figure 2), displaying a trend toward a higher risk. Re‐hospitalization did not significantly differ but showed a trend toward an increase in digitalis‐treated patients (45.8% vs. 41.6%; *p* = 0.074). Shock delivery was also reported more often in digitalis‐treated patients (19.7% vs. 15.0%, *p* = 0.006). Device revision was also performed more frequently in digitalis‐treated patients (11.4% vs. 7.5%, *p* = 0.004, Table 4).

**TABLE 4:** In‐hospital complications and complications during follow‐up.

	Digitalis group	Control group	*p*
In‐hospital complications			
Death [%]	0	0.3 (*n* = 10)	0.13
Nonfatal stroke [%]	0.3 (*n* = 2)	0	0.052
Nonfatal myocardial infarction [%]	0	0	1
Device revision [%]	0.9 (*n* = 6)	2.5 (*n* = 57)	0.013
Complications during follow‐up			
Mortality^a^ [%]	9.1	7.4	0.14
MACCE (death, MI or stroke)^a^ [%]	10.4	8.4	0.08
Re‐hospitalization [%]	45.8 (*n* = 247)	41.6 (*n* = 905)	0.074
ICD shock delivery [%]	19.7 (*n* = 109)	15.0 (*n* = 333)	0.006
Device revision [%]	11.4 (*n* = 60)	7.5 (*n* = 158)	0.004

Abbreviations: MACCE = death, myocardial infarction, stroke, MACE = death, myocardial infarction.

^a^ Kaplan–Meier estimates at 366 days after index discharge.

Of note, the prevalence of atrial fibrillation at baseline was significantly higher in the digitalis group (35.9% vs. 16.3%, *p* < 0.001). A consecutively performed regression analysis identified this factor as a major determinant for increased mortality and ICD shock deliveries (Table 5).

**TABLE 5:** Implantation procedure and discharge data.

	Digitalis group	Control group	*p*
Duration of surgery [min]	57	69	0.54
Defibrillation test performed [%]	72.8 (*n* = 550)	74.5 (*n* = 2120)	0.37
Pericardial effusion [%]	0.1 (*n* = 1)	0.2 (*n* = 5)	0.58
Pneumothorax [%]	0.1 (*n* = 1)	0.5 (*n* = 16)	0.23
Pocket hematoma [%]	1.5 (*n* = 12)	1.3 (*n* = 39)	0.61
Wound complication [%]	0.6 (*n* = 5)	0.4 (*n* = 11)	0.35

Assessment of patient satisfaction reflected that the majority of patients in both groups considered treatment as successful (90.3% vs. 92.2%; *p* = 0.38) and would again decide to undergo treatment (97.1% vs. 94.0%, *p* = 0.54). Of note, patients with digitalis therapy reported significantly more often that they were afraid of potential shock delivery (*p* = 0.043).

## 4 Discussion

This study reports “real‐world” data on the clinical employment of digitalis glycosides in patients undergoing ICD and CRT systems. The proportion of patients receiving digitalis glycosides is about 20% of the whole population. In the presence of more nonischemic cardiomyopathy and more comorbidities in the digoxin group, digitalis glycosides were associated with an increased risk for rehospitalization or device revision while no significant differences in mortality or major complications, including stroke or myocardial infarction, were observed. Nonetheless, a trend toward an increased risk was observed in digitalis‐treated patients. Comparable results have been reported in a large observational study in CRT‐recipients (Erath et al. 2024) and in a large single center report in ICD‐patients (Erath et al. 2016) as well as in the MADIT‐CRT trial where no significant impact on mortality by digoxin but an increased risk for ventricular arrhythmias was reported (Lee et al. 2015).

### 4.1 Patient Collective and Demographics

The present patient cohort represents a typical collective of patients undergoing cardiac device implantations. Observed differences between digitalis‐treated and control patients were observed regarding cardiovascular disease. Dilated cardiomyopathy and atrial fibrillation were more common in digitalis‐treated patients who have worse LV function and more severe heart failure. Thus, CRT systems were chosen more frequently in digitalis‐treated patients. It is likely that the increase in hospitalization can solely be explained by the differences in patient characteristics. However, other previous trials suggested a potentially elevated risk of adverse events with digoxin (Turakhia et al. 2014; Hallberg et al. 2007; Whitbeck et al. 2013) although these observations have also not been directly attributed to digitalis treatment itself (Kirchhof et al. 2016). In a post hoc propensity‐matched analysis of the AFFIRM trial, no evidence of increased mortality or hospitalization was reported in patients taking digoxin as baseline initial therapy (Gheorghiade et al. 2013).

### 4.2 Procedural Data and Follow‐Up

Periprocedural complications did not significantly differ between both study groups. Overall, the incidence of severe and other complications was low. During follow‐up, no significant differences in mortality or severe cardiovascular events were observed. Nonetheless, a trend toward a higher risk in digitalis‐treated patients was noted. However, shock deliveries were more common in digitalis‐treated patients. This aspect is probably explained by the fact that severe heart failure was more common in the digitalis group. This is reflected by significant differences in LVEF and NYHA class and also an increased proportion of CRT systems in the digitalis group.

Furthermore, the risk for a device revision was significantly elevated in digitalis‐treated patients. This observation may be explained by the fact that the percentage of more complex systems was higher in digitalis‐treated patients where diabetes as comorbidity was also more prevalent. Consequently, the risk for electrode dysfunction or hematoma may have been elevated in this group.

Digitalis therapy itself could also possibly exert negative effects because of a certain risk of proarrhythmic events. This idea is not new and has recently been in particular evaluated for the combination of digitalis glycosides with antiarrhythmic agents like dronedarone (Hohnloser et al. 2014; Frommeyer et al. 2015). However, a direct proarrhythmic effect cannot be proven in the present study.

## 5 Limitations

The design of the registry may include a certain selection bias as patient selection cannot be as precise as in randomized clinical trials. The prescription of digitalis glycosides usually depends on relevant comorbidities and additional undocumented factors. Whether digoxin or digitoxin was employed was not differentiated in the present registry. In addition, information on serum concentrations was not obtained. As the analysis of complementary data is not as thorough as in randomized trials, the results of this study ought to be interpreted in a descriptive and hypothesis‐generating sense. In addition, follow‐up duration is rather short, which is due to the design of this registry, and specific information on device revisions during follow‐up was not available. Nonetheless, the results of this study represent “real‐life” data on patients receiving digitalis therapy and report important insights on patient characteristics and outcomes.

## 6 Conclusion

In this study, no differences in mortality or severe cardiovascular events were observed between digitalis‐treated patients and patients without digitalis therapy. This is an important difference as compared to previous reports in different patient cohorts such as the German Ablation Registry (Frommeyer, Brachmann, et al. 2019). However, an increased risk of ICD shock delivery and of device revision was observed in digitalis‐treated patients that may be explained by different patient characteristics. As suggested in previous studies, it cannot be distinguished whether digitalis therapy promotes a poorer prognosis or may just serve as a marker for an increased disease burden.

## Author Contributions

Conception and Design: G.F., J.S., L.E. Drafting: G.F: Data acquisition: M.H., Critical Revision and final approval: T.K., C.S., H.I., J.B., T.L., L.E.

## Conflicts of Interest

G.F. received speaker's honoraria from Abbott, Boston Scientific, and Pfizer Pharma as well as research grants from Abbott, Biosense Webster, Biotronik, Boston Scientific, Daiichi Sankyo, and Pfizer Pharma. J.B. discloses advisory board activities for iRhythm and Johnson & Johnson. T.L. reports lecture honoraria from Biotronik, Boston Scientific, Medtronic, and Abbott. L.E. discloses consultant fees, speaking honoraria, and travel expenses from Abbott, Bayer Healthcare, Biosense Webster, Biotronik, Boehringer, Boston Scientific, Bristol‐Myers Squibb, Daiichi Sankyo, Medtronic, Pfizer, and Sanofi Aventis.

## Data Availability

Research data are not shared.
